# Experimental Sensing and Density Functional Theory Study of H_2_S and SOF_2_ Adsorption on Au‐Modified Graphene

**DOI:** 10.1002/advs.201500101

**Published:** 2015-09-10

**Authors:** Xiaoxing Zhang, Lei Yu, Xiaoqing Wu, Weihua Hu

**Affiliations:** ^1^ State Key Laboratory of Power Transmission Equipment and System Security and New TechnologyChongqing UniversityNo.174 in Shazheng StreetShapingba DistrictChongqing400044P.R. China; ^2^ School of Electrical EngineeringWuhan UniversityNo.16 in Luojiashan Road, Wuchang DistrictWuhanHubei Province430072P.R. China; ^3^ Institute for Clean Energy and Advanced MaterialsSouthwest UniversityChongqing400715P.R. China

**Keywords:** adsorption, Au‐modified graphene, DFT calculations, SF6 decomposed gas, surface interaction

## Abstract

A gas sensor is used to detect SF_6_ decomposed gases, which are related to insulation faults, to accurately assess the insulated status of electrical equipment. Graphene films (GrF) modified with Au nanoparticles are used as an adsorbent for the detection of H_2_S and SOF_2_, which are two characteristic products of SF_6_ decomposed gases. Sensing experiments are conducted at room temperature. Results demonstrate that Au‐modified GrF yields opposite responses to the tested gases and is thus considered a promising material for developing H_2_S‐ and SOF_2_‐selective sensors. The first‐principles approach is applied to simulate the interaction between the gases and Au‐modified GrF systems and to interpret experimental data. The observed opposite resistance responses can be attributed to the charge‐transfer differences related to the interfacial interaction between the gases and systems. The density of states and Mulliken population analysis results confirm the apparent charge transfer in Au‐modified GrF chemisorption, whereas the van der Waals effect dominates the pristine graphene adsorption systems. Calculation results can also explicate the significant SOF_2_ responses on Au‐modified GrF. This work is important in graphene modulation and device design for selective detection.

## 1 Introduction

Sulfur hexafluoride (SF_6_) is widely used in gas‐insulated electrical equipment because it features excellent insulating and arc‐extinguishing properties. At the early stages of insulation degradation of electrical equipment, the occurrence of partial discharge (PD) causes the decomposition of SF_6_ into various products, such as H_2_S, SO_2_, SOF_2_, and SO_2_F_2_.^1^ These products can accelerate PD evolution in reverse, resulting in sharp reduction in the insulation strength of electrical equipment and formation of insulation faults. The composition and content of these decomposed components must be determined to identify the types, severities, and factors affecting PD and accurately assess the insulation status of electrical equipment.^2^, ^3^, ^4^, ^5^ Gas sensors are used to detect the composition and content of SF_6_ decomposed gases.^6^

Graphene is a promising material for various applications, such as energy storage,^7^ nanoelectronic devices,^8^ fuel cells,^9^ and capacitors,^10^ because it features very high electron mobility caused by linear dispersion relation in the band structure of the π and π^*^ states around the Dirac point in the Brillouin zone.^11^ Graphene sheets have been increasingly used as transducers since the first successful attempt to apply graphene for chemical sensing and detection of single‐gas molecular absorption and desorption on graphene platforms.^12^ Experimental and theoretical studies have been conducted to elucidate the related gas adsorption mechanism. Nevertheless, previous studies mainly focused on common gaseous molecules, such as NH_3_,^13^ NO_2_,^14^, ^15^ and CO_2_.^16^

Graphene sheets are functionalized with metals, chemical treatments, or other modifiers to enhance the chemisorption and selectivity of graphene to target gases.^17^, ^18^ Doping metallic nanoparticles is a practical method for tuning the electronic structure of graphene sheets without significantly losing mobility. The reduced graphene oxide (rGO) sensors doped with Pd by spin coating,^19^ layer‐by‐layer (LBL) deposition,^20^ or chemical vapor deposition^21^ are used as functional films for hydrogen detection. Density functional theory (DFT) calculations further revealed that metal embedded and structural defects on graphene can effectively tune the electron density and transport properties of this carbon. In particular, Au‐ and Fe‐embedded graphene have been used to enhance the chemical reactivity of graphene for potential applications in catalysts and gas sensing.^22^

In this study, we used Au‐modified graphene films (Au‐modified GrF) as gas sensors for the detection of H_2_S and SOF_2_. Low‐cost and simple LBL deposition was adopted in device fabrication to investigate H_2_S and SOF_2_ adsorption responses. Based on first‐principles calculations, we studied the interaction mechanism of the Au‐modified GrF substrate with the gases. To the best of our knowledge, theoretical study combined with experimental data regarding the SOF_2_ adsorption effect on Au‐modified GrF has not been conducted and a thorough study of H_2_S adsorption effect is rarely reported.

## 2 Results and Discussion

### 2.1 Experimental Sensing Performance

**Figure 1:**
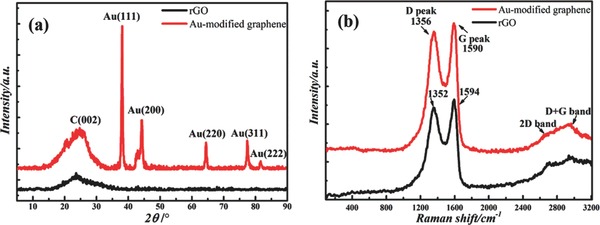
a) XRD patterns and b) Raman spectra of rGO and Au‐modified GrF.

Materials must be thoroughly analyzed before the investigation of sensing performance. **Figure** **1**a presents the XRD patterns of Au‐modified GrF and rGO. The Au characteristic peaks in Au‐modified GrF are located at 38.1° (111), 44.3° (200), 64.5° (220), 77.55° (311), and 81.65° (222), whereas the standard Au peaks at 38.184° (111), 44.392° (200), 64.576° (220), 77.547° (311), and 81.721° (222). The main Au (111) peak suggests the formation of a crystal phase, and the broad Au peaks imply that Au nanoparticles (AuNPs) are highly dispersed in the sample. The particle size of 14 nm was estimated from the main peak width of Au (111) through the Scherrer formula. The broad diffraction peaks at 24.63° and 23.77° for Au‐modified GrF and rGO samples, respectively, reveal the carbon structure of graphene.

Raman spectra were determined for structural characterization.^23^ The typical postsubtraction Raman spectra were directly recorded on the sensor substrate and shown in Figure 1b. As the epoxy layers underlying the films function as insulators, the effect on electron distribution is disregarded. The comparison of the Raman spectra between Au‐modified GrF and pure rGO films indicates that low amounts of modified AuNPs do not significantly change the formation of the in‐plane sp^2^ domains in graphene. The Raman spectra of the films show the representative peaks at ≈1356 (D) and ≈1590 cm^−1^ (G) and the broad bands between 2400 and 3200 cm^−1^.^20^ As the intensity ratios of the D and G peaks are highly sensitive to the quality of material,^24^ we speculate that graphene‐based materials contain multiple layers. Furthermore, the D peak is relatively pronounced, indicating the presence of a significant number of defects and a degree of confusion structure caused by deprivation of oxygen functionalities during hydrogen reduction.^25^ The sample flakes present a size of tens nanometers based on the D/G ratio of 0.85 and the G peak position. This observation is consistent with the results of XRD analysis.

**Figure 2:**
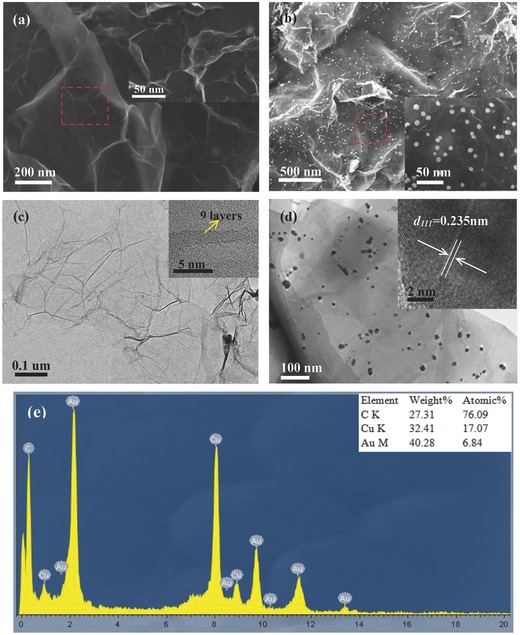
a) SEM image of rGO, b) SEM image of Au‐modified GrF, c) TEM and HRTEM images (inset) of rGO, d) TEM and HRTEM images (inset) of Au‐modified GrF, and e) EDS spectrum of Au‐modified GrF.

**Figure** **2**a shows low‐ and high‐resolution SEM images of pure rGO. The clear and bright vision of rGO film sample indicates good electrical conductivity, which is free of metal spraying treatment. The flimsy, wrinkled, and fluctuant surface of two‐dimensions is the typical morphology of rGO in practice application. Morphological structure, particle size, and metal dispersion of Au‐modified GrF were also determined and the results are illustrated in Figure 2b. The enhanced area caused by highly dispersed AuNPs provides more available active sites on the sensing surface. AuNPs are uniformly embedded and covered on rGO. After analyzing several images, the average diameter of AuNPs is determined as tens of nanometers; this finding is in agreement with the results of XRD and Raman studies. Figure 2c,d shows the TEM morphologies of pure rGO and Au‐modified GrF, respectively. The rGO sample is with good light transmittance and obvious fold boundary. These features indicate that the quality of rGO sample is excellent, which owns few carbon layers. The specific number regarding these carbon layers is further confirmed to nine by HRTEM, which is embedded in Figure 2c. Therefore, our rGO sample is classified as the multilayer graphene. From the TEM image in Figure 2d, we confirm that AuNPs with dozen nanometer sizes are homogenously dispersed on the rGO sheet surface. The HRTEM images in the inset in Figure 2d reveal the well‐defined lattice fringes of Au (111) with a clear lattice distance (*d*_111_ = 0.235 nm), indicating that AuNPs exist and are highly crystalline. The EDS spectrum further verifies that Au is successfully modified in graphene.

**Figure 3:**
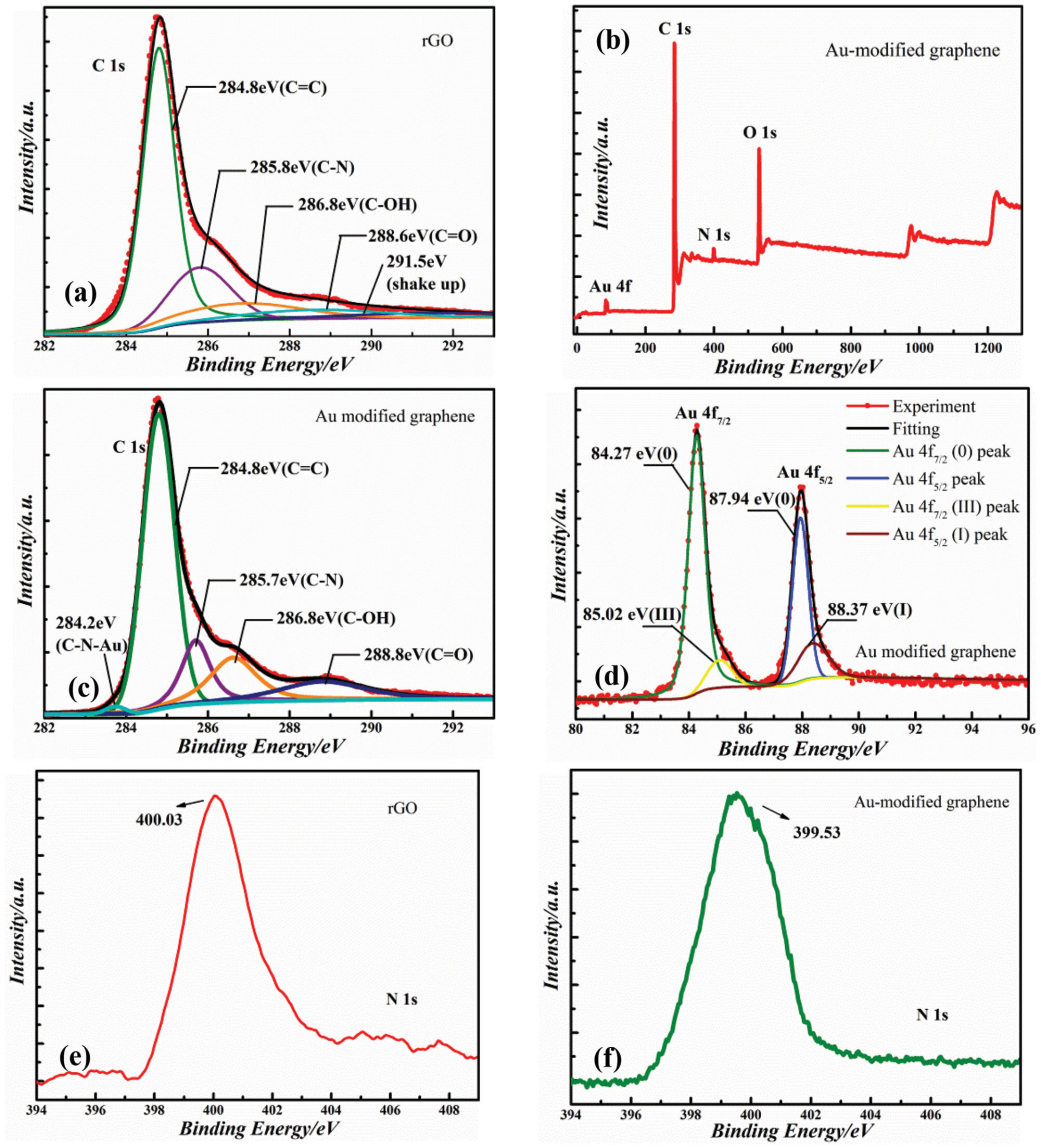
XPS spectra of a) C 1s region of rGO, b) Au‐modified GrF, c) C 1s region of Au‐modified GrF, d) Au 4f region of Au‐modified GrF, e) N 1s region of rGO, and f) Au‐modified GrF.

XPS spectra are important in valence analysis. **Figure** **3** shows the XPS spectra of rGO and Au‐modified GrF. The results reveal that ≈6% AuNPs are successfully modified on the graphene surface, which is in accordance with the XRD, SEM, and TEM results. The high‐resolution C 1s XPS spectrum of rGO indicates that numerous heteroatom defects exist on the plane and edges.^26^ Moreover, the C 1s spectrum can be fitted into four peaks corresponding to C atoms from four functional groups: nonoxygenated ring C at 284.8 eV, C in the C—N bond at 285.8 eV, hydroxyl C at 286.8 eV, and carbonyl C at 288.6 eV. After Au was introduced, a new peak appears at 284.2 eV and this peak is attributed to the C—N—Au bond.^26^, ^27^, ^28^ After peak‐differentiation‐imitating analysis, the Au 4f doublet deconvolutes into two pairs of peaks, which correspond to the reduced Au (0) at 84.27 eV in Au 4f_7/2_ and at 87.94 eV in Au 4f_5/2_, Au (III) ions at 85.02 eV in Au 4f_7/2_ and Au (I) ions at 88.37 eV in Au 4f_5/2_, respectively.^29^ Approximately 0.6 eV redshift of the XPS peak of Au (0) ions exists in Au 4f_7/2_. This shift in the bonding energy may be ascribed to the substrate and reduced core‐hole screening in metal particles. Furthermore, a dynamic electron transfer from modified AuNPs to the supported graphene films is confirmed by the existence of positively charged Au (III) and Au (I) ions according to the electrostatic balance principle and theoretical calculation in DFT study. Moreover, according to the N 1s spectra of rGO and Au‐modified sample in Figure 3e,f, modified AuNPs result in a blue shift of binding energy (from 400.03 eV to 399.53 eV), which indicates that Au‐modified sample is a covalent hybrid based on the coordination or chemical effects between heteroatoms, such as, N and O, and Au clusters.^26^, ^30^, ^31^

**Figure 4:**
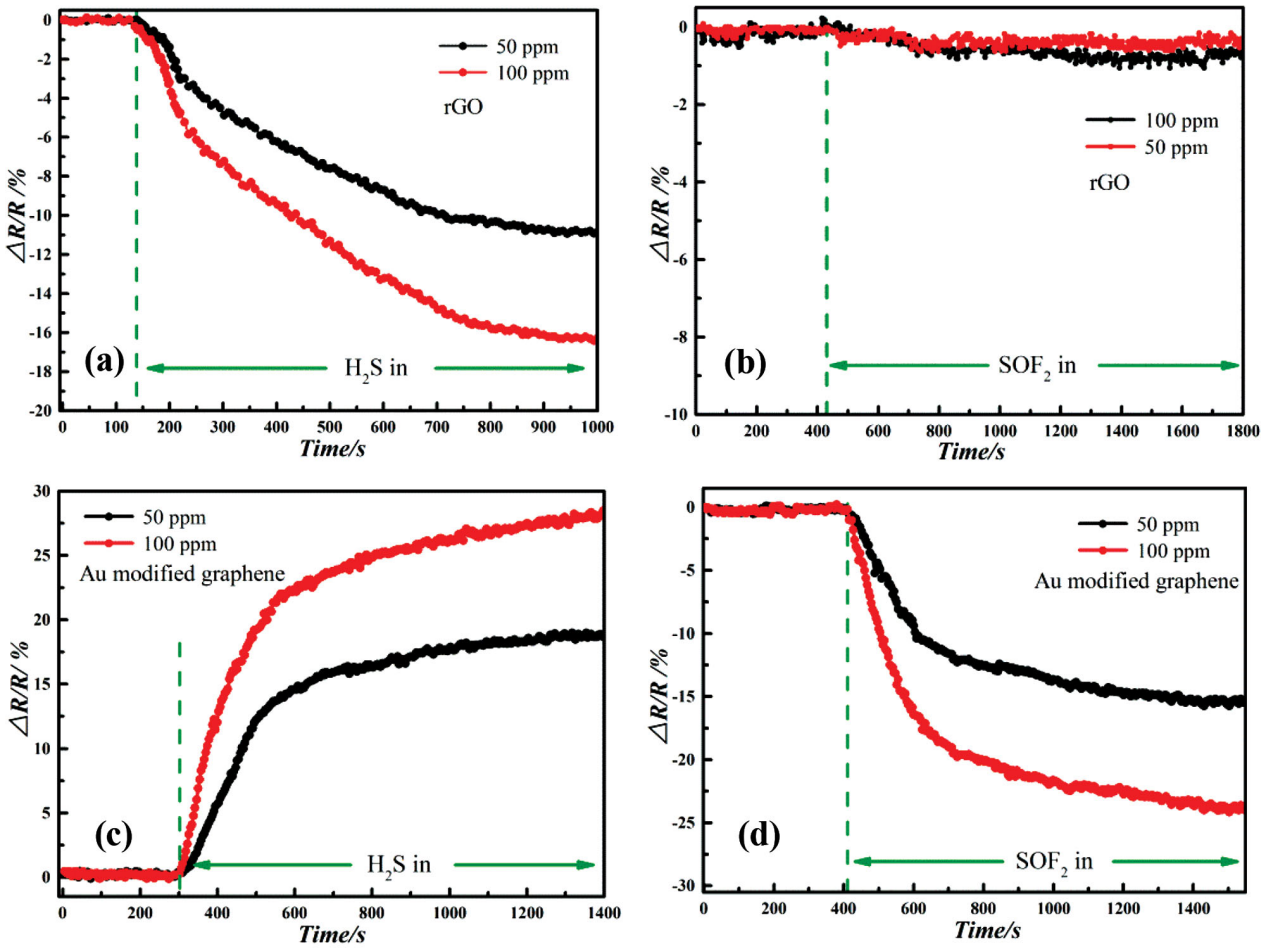
Experimental transient responses of rGO sensors to a) H_2_S and b) SOF_2_, and Au‐modified GrF sensors to c) H_2_S and d) SOF_2_.

Target gas molecules, namely, H_2_S and SOF_2_, were delivered to the graphene‐based sensor device through mass flow‐controlled dilution with clean, pure, and dry helium. **Figure** **4**a–d represents the responses of the rGO and Au‐modified GrF sensors in terms of resistance changes during exposure to varied concentrations of target gases in an autonomous sealed chamber. Resistance change is defined as

(1) $$\Delta R=R_{F}-R_{I}$$

where *R*_F_ represents the sensor resistance at the final target gas exposure and *R*_I_ represents the initial vacuum resistance at the previous rest period. Sensor response^32^ is universally defined as

(2) $$\mathrm{Sensitivity}=\frac{\Delta R}{R_{I}}\times 100\%$$

Although humidity and temperature significantly affect the sensor response, specific operating conditions in electrical equipment may reduce this variation. Figure 4a–d shows the sensor responses after exposure to 50 and 100 ppm of H_2_S and SOF_2_. Only the responses associated with these two concentrations were analyzed to investigate the sensing mechanism. For rGO under ambient conditions, 100 and 50 ppm H_2_S cause 15.78% and 10.53% reduction in resistance, respectively. SOF_2_ is not sensitive to rGO. Moreover, 100 and 50 ppm SOF_2_ result in 23.83% and 15.36% reduction in the resistance of Au‐modified GrF, respectively. These findings indicate that Au‐modified GrF exhibits higher sensitivity to SOF_2_ than rGO. Although Au‐modified GrF is also sensitive to H_2_S, a positive resistance change was observed. In this system, 100 and 50 ppm H_2_S increase the resistance by 28.15% and 18.73%, respectively. These sensing performances reveal that the Au‐modified GrF and pure rGO sensors are promising materials for selective detection. Therefore, the sensing behavior was further analyzed.

**Figure 5:**
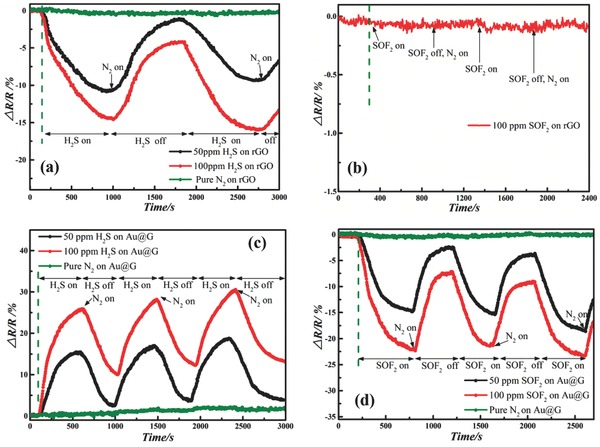
Transient responses of rGO sensors to a) H_2_S, b) SOF_2_ flows, and Au‐modified GrF sensors to c) H_2_S, d) SOF_2_ flows.

Further experiments were performed to determine the response properties under repeated gas pulses. For this, a series of sensing experiments involving H_2_S and SOF_2_ were performed at the same setting. **Figure** **5**a,b shows our rGO sensor's behavior in repeated H_2_S and SOF_2_ flows, respectively, and Figure 5c,d illustrates the responses of our Au‐modified GrF sensor to target gases. The target gas and pure N_2_ were flown over the sensor sequentially and recurrently, and the changes in resistance were measured. N_2_ flow expelled the target gas that had already been existed, for the purpose of exploring its recovery property and preparing the next detection round at the same time. Based on the results in Figure 5, the sensor response presents the similar variation tendency in comparison with the single test result, regardless of the magnitude and response direction. In addition, we have noticed that the transient for H_2_S on rGO sensor is much slower compared to the response behavior on Au‐modified GrF. Hence, the response speed of Au‐modified GrF is proved to be better than pure rGO.

### 2.2 DFT Study of Adsorption Mechanism

#### 2.2.1 Au‐Modified Graphene

**Figure 6:**
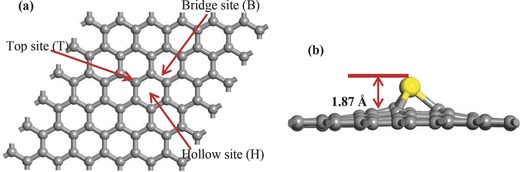
a) Three possible modification positions of Au atom on graphene. b) Side view of partial atomic structure for T site modification model.

**Figure 7:**
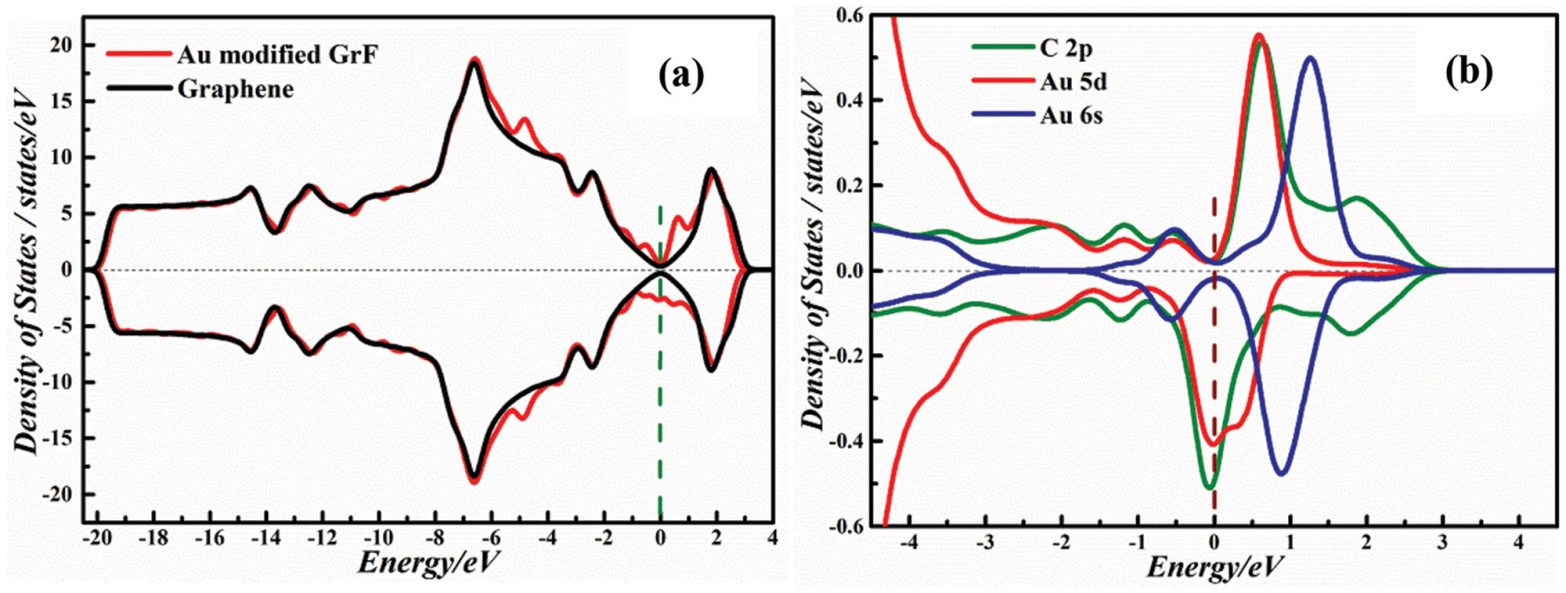
a) Spin‐polarized DOS of pristine graphene (black curve) and Au‐modified GrF (red curve) and b) PDOS projected on the s orbital (blue curve) and d orbital (red curve) of Au and 2p orbital (green curve) of neighboring C.

A simulation model was constructed for theoretical calculation to investigate the factors affecting sensor performance and the sensing mechanism. We already discussed the nature of the bonding between decorated Au and graphene. The three possible positions^22^ considered are the top site above a carbon atom (T), bridge site between the C—C bond (B), and hollow site at the hexagon center (H), which are illustrated in **Figure** **6**a. Our calculation results show that the T site model holds the lowest energy, as shown in Figure 6b. And the detailed calculated energy information has been summarized in Table S1 (Supporting Information). Therefore, our calculation focuses on the T position, in which Au substitutes C. Au atoms in Au‐modified GrF adopt a partial sp^3^ configuration and protrude from the graphene plane by 1.87 Å along the Z axis. The bond lengths (2.07 Å) of the three C—Au bonds confirm the partial sp^3^ configuration of Au atoms. A similar bonding geometry was observed in *M*‐modified graphene (*M* = Pd, Pt, and Mn)^33^, ^34^, ^35^ from previous PBE calculations. In the present calculation, the electrons on Au are depleted when Au bonded to C atoms. This observation is in agreement with the presence of positively charged Au (I) and Au (III) ions in the XPS analysis. The spin‐polarized density of states (DOS) of Au‐modified GrF and pristine graphene are illustrated in **Figure** **7**a. Au instigates finite DOS and introduces an evident conductance change near the Fermi level to pristine graphene, indicating that the presence of Au endows the zero‐gap material with a metallic property. Moreover, Au breaks the symmetry between spin‐up and spin‐down channels and generates a magnetic moment (−1.005 μ_B_). According to the partial DOS (PDOS) in Figure 7b, magnetic moments mainly originate from the depleted spin‐down electrons of the d orbital of Au and the p orbital of the neighboring C.

#### 2.2.2 Gas Adsorption Effects

**Figure 8:**
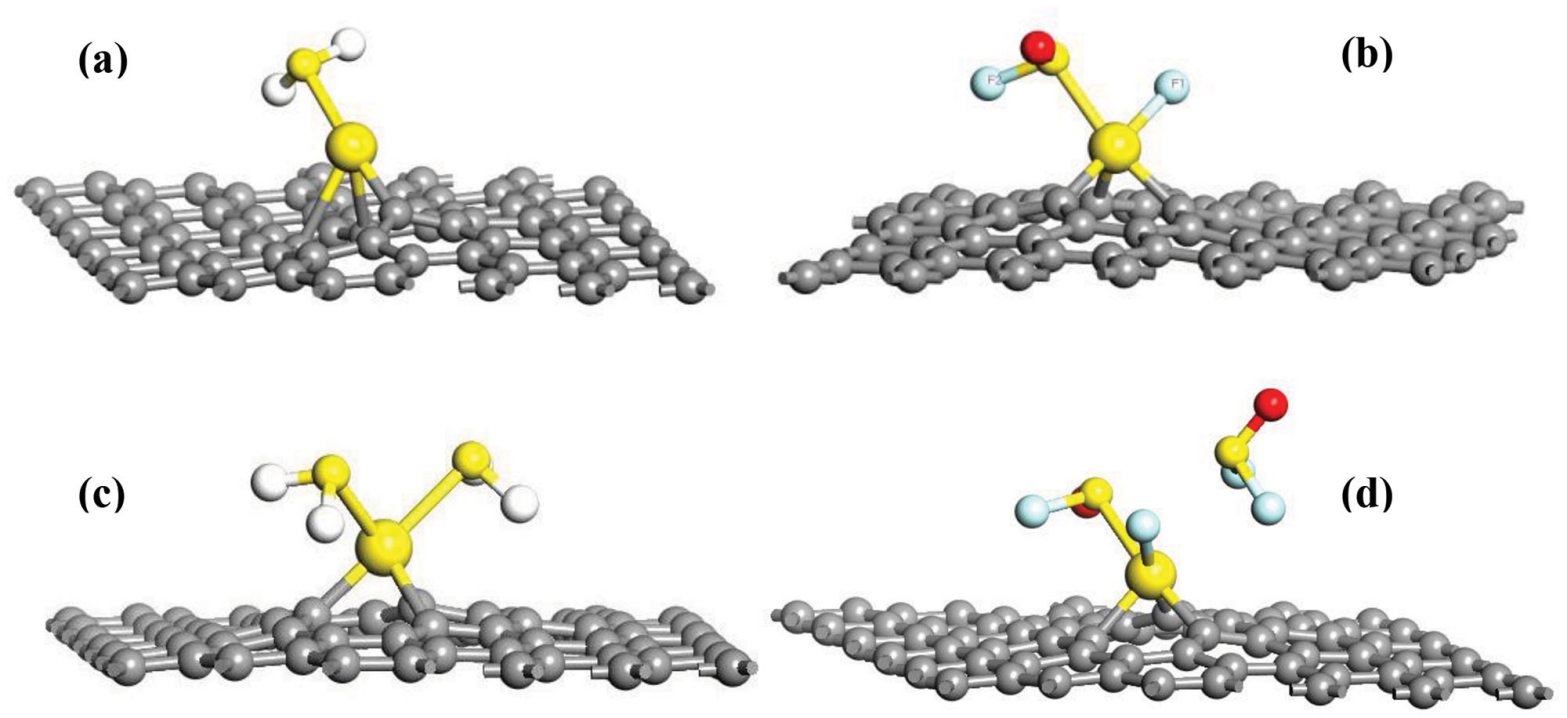
Optimized configurations of single a) H_2_S, b) SOF_2_, c) 2H_2_S, and d) 2SOF_2_ adsorbed on Au‐modified GrF.

**Figure 9:**
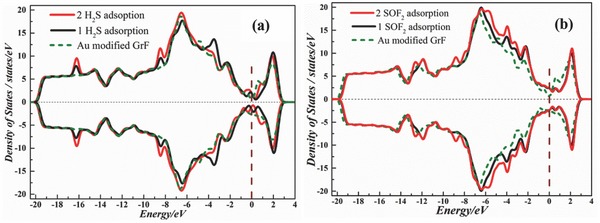
a) Spin‐polarized DOS of Au‐modified GrF (dash olive curve), Au‐modified GrF‐H_2_S (solid black curve), and Au‐modified GrF‐2H_2_S (solid red curve). b) Spin‐polarized DOS of Au‐modified GrF (dash olive curve), Au‐modified GrF‐SOF_2_ (solid black curve), and Au‐modified GrF‐2SOF_2_ (solid red curve). The Fermi energy is set as *E − E*_f_.

Different orientations are required to achieve the most stable adsorption configuration, which exhibits the lowest total energy and highest adsorption energy.^36^ **Figure** **8** illustrates the most stable adsorption configurations of single and double H_2_S and SOF_2_ molecules on Au‐modified GrF. **Table** **1** presents the calculated results of every adsorption system. **Figure** **9** further shows the spin‐polarized DOS for H_2_S and SOF_2_ adsorption systems. We focused on the adsorption effects of double molecules on the Au‐modified GrF plane according to previous research, which reported that several molecule adsorption cases were inclined to be unstable at room temperature because of significant decreases in their *E*_ad_.^22^ Although part of Au concentration was ignored in DFT calculation, we aimed to establish the basic principle by using a simple model. The influence of Au concentration on the mechanisms underlying sensing properties was further investigated.

**Table 1:** Adsorption parameters in single‐ and double‐target molecule cases

Configuration	*d*_1_ a) [Å]	*d*_2_ a) [Å]	*E*_ad_ b)[eV] (with DFT‐D)	*Qt*_1_ c) [*e*]	*Qt*_2_ c) [*e*]
Graphene‐H_2_S	3.108		−0. 617	+0.011	
Au‐modified GrF‐H_2_S	2.401		−0.900	+0.348	
Au‐modified GrF‐2H_2_S	2.593	2.598	−1.718	+0.304	+0.302
Graphene‐SOF_2_	3.526		−0.267	−0.002	
Au‐modified GrF‐SOF_2_	2.039		−0.961	−0.624	
Au‐modified GrF‐2SOF_2_	2.519	3.970	−1.334	−0.658	−0.031

^a)^ *D* denotes the shortest distance between the molecule and the oriented substrate, where *d*_1_ and *d*_2_ represent former and latter molecules, respectively;

^b)^ *E*_ad_ describes the surface interaction *E*_molecule/AuNPs@graphene_ − *E*_molecule_ − *E*_AuNPs@graphene_;

^c)^ *Qt* represents the net charge transformation, which indicates the redistribution charges in the adsorption system, where *Qt*_1_ and *Qt*_2_ refer to former and latter molecules, respectively.

*H_2_S Adsorption Cases*: In the Au‐modified GrF‐H_2_S system, H_2_S is adsorbed parallel to the surface with S atoms closest to Au. The geometric structure of H_2_S slightly changes as the H—S—H bond angle (91.181°) expands to 91.719° and the H—S bond angle (1.356°) extends to 1.360 and 1.362 Å. H_2_S molecules also prefer a closer configuration on the Au‐modified GrF plane than that on the pristine graphene, as indicated by the shorter distance between Au‐modified graphene and S (2.401 Å) than that between pristine graphene and H_2_S (3.108 Å). Hence, H_2_S molecules are chemisorbed on Au‐modified GrF as evidenced by the high adsorption energy (−0.9 eV) and electron transfer (0.348 *e*) from H_2_S molecules to Au‐modified GrF. This electron transfer leads to electron enrichment on the Au‐modified GrF surface. In graphene‐H_2_S adsorption case, the obtained electron transfer value (0.011 *e*) indicates the presence of few electron interactions. Nevertheless, the adsorption energy of graphene–H_2_S (−0.617 eV) presents a relatively strong intermediate between physisorption and chemisorption,^37^, ^38^ which indicates the prevalence of van der Waals. By comparing the studies on Au‐modified GrF‐H_2_S and pure Au‐modified GrF, we observed an evident difference in DOS near the Fermi level, indicating a decrease in the surrounding DOS. For Au‐modified GrF, the Fermi level shifts upward by 0.043 eV, which is reminiscent of an n‐type effect caused by adsorbed H_2_S molecules. The magnetic moment further changes to 0.999 μ_B_.

We found that the two H_2_S molecules exhibiting a similar configuration are the stable configuration on Au‐modified GrF; in this configuration, each S atom moves closer to Au. The binding geometry is similar to that of the Au‐modified GrF‐H_2_S system. The adsorption energy (−1.718 eV) is two times higher than that of Au‐modified GrF‐H_2_S. Although ≈0.3 electrons per H_2_S molecule transfer to the Au‐modified GrF complex in the Au‐modified GrF‐2H_2_S system, the system still exhibits an n‐type sensing nature. The n‐type effect introduced by the two H_2_S cases could be attributed to the upward shift of the Fermi level by 0.077 eV. This observation is also consistent with the higher adsorption energy of 2H_2_S on Au‐modified GrF than 1H_2_S. In fact, our separate PDOS calculation confirms that Au prefers to hybridize S atoms in the H_2_S valence band, which contributes to the strong interaction between Au and adsorbed H_2_S. A comparison study using Figure 9a suggests that 2H_2_S adsorption remains magnetic in the semiconductor system with a magnetic moment of 0.823 μ_B_.

*SOF_2_ Adsorption Cases*: In the Au‐modified GrF‐SOF_2_ case, SOF_2_ prefers the configuration in which SOF_2_ is located above Au‐modified GrF; in this configuration, S and F atoms bond to Au as indicated by the distance between S and Au (2.508 Å) and F and Au (2.039 Å). The F—S bond extends longer (2.894 Å) than that in the gas phase (1.668 Å) because of the charge transfer, indicating SOF_2_ rupture. The results of Mulliken population analysis confirm the occurrence of a significant electron transfer (0.624 *e*) from Au‐modified GrF to SOF_2_; this phenomenon features a p‐type process with SOF_2_ as an acceptor. The adsorption energy (−0.961 eV) is similar to that in the Au‐modified GrF‐H_2_S case. Our DOS calculation results further reveal that the spin‐up and spin‐down channels shift downward by 1.05 eV, indicating the strong p‐type effect of SOF_2_ adsorption. The adsorbed SOF_2_ converts the system of Au‐modified GrF from magnetic metal into a nonmagnetic system with magnetic moment quenching. This observation differs from that in the H_2_S cases, which maintains the magnetic property.

When two SOF_2_ molecules move closer to Au‐modified GrF, the most stable configuration is where one SOF_2_ prefers the active state and approaches the Au‐modified GrF plane and the other SOF_2_ moves away from Au‐modified GrF (Figure 8d). The inactive SOF_2_ does not effectively participate in the interaction process as also confirmed by the results of Mulliken population analysis. Approximately 0.658 electrons transfer to the active SOF_2_, whereas 0.031 electrons transfer to the inactive site. Minimal change is introduced to the DOS around the Fermi level (the overlapping solid black and red curves) because of the inactive SOF_2_. In the H_2_S case, this observation differs from that in 2H_2_S adsorption, in which one H_2_S exhibits equivalent effect to the other H_2_S. The adsorption energy of the Au‐modified GrF‐2SOF_2_ system (−1.334 eV) is higher than that in Au‐modified GrF‐SOF_2_ (0.961 eV). The small amounts of additional energy can be ascribed to the van der Waals interaction, which is supplemented by electrostatic effect caused by chemically inactive SOF_2_ molecules. The results of DOS analysis further indicate that the Fermi level shifts downward by 0.966 eV compared with that of Au‐modified GrF; this phenomenon demonstrates a p‐type effect. Moreover, Au‐modified GrF‐2SOF_2_ case maintains its nonmagnetic property similar to the Au‐modified GrF‐SOF_2_ system.

#### 2.2.3 Discussion of Sensing Performances Based on DFT Calculations

Figure 4a,c presents the responses of H_2_S, which is significantly sensitive to rGO and Au‐modified GrF but exhibits opposite effect compared with each other. As confirmed in our DFT calculations, the pronounced chemisorption effect contributes to the H_2_S responses on Au‐modified GrF. However, the results also prove that the adsorption interaction with pure graphene is typical physisorption, which contradicts the experimental findings. The factors affecting this discrepancy remain unclear, but we postulate that it could be related to the method used to manufacture pure graphene films. The pure graphene films used in this study were prepared through chemical reduction, which could inevitably harbors heteroatoms, such as N and O. The combined XPS analysis results and Raman spectra reveal the presence of nonoxygenated ring, C—N bonds, hydroxyl C, and carbonyl, which may affect the sensing interaction. This phenomenon must be further investigated through theoretical and experimental studies.

For SOF_2_, sensing experiments were conducted on pure graphene and Au‐modified GrF (Figure 4b,d) to investigate the influence of decorated Au on gas sensing. The sensing performance of SOF_2_ on pure graphene is not significant as it only results in 0.7% decrease in resistance. This response is significantly lower than that of Au‐modified GrF, which decreases the resistance by a maximum of 23.83%. In DFT calculations, the interaction between SOF_2_ and Au‐modified GrF is chemisorption as evidenced by the charge transfer from Au‐modified GrF to SOF_2_ (0.624 *e* in the Au‐modified GrF‐SOF_2_ system). This finding could also be attributed to the higher chemical potentials of Au‐modified GrF than the lowest unoccupied molecular orbital of SOF_2_. Nevertheless, as SOF_2_ is physisorbed on the pure graphene surface, no charge transfer (Table 1) could occur. This physisorption phenomenon, in which only the van der Waals effect dominates, could contribute to the unclear responses of SOF_2_ on pure graphene. In addition, the calculated adsorption energy (−0.267 eV) is not significant in the graphene‐SOF_2_ system compared with that in the Au‐modified GrF‐2SOF_2_ system (−1.334 eV). Therefore, based on our experiment and calculation results, we successfully established an effective approach to manufacture SOF_2_ sensors.

**Figure 10:**
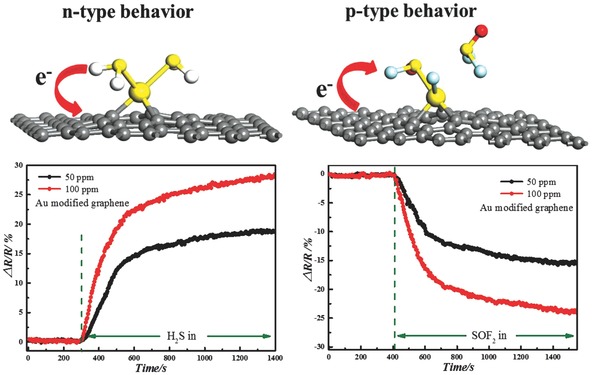
Correlation between sensing performance and nature of carriers for Au‐modified GrF.

Figure 4c,d presents the comparison between the performance of a resistive sensor with H_2_S and the SOF_2_ sensing results obtained using Au‐modified GrF. Au‐modified graphene exhibits a 28.15% increase in resistance for 100 ppm H_2_S and 23.83% decrease in resistance after 12 min of exposure to 100 ppm SOF_2_. The fabricated Au‐modified graphene sensor demonstrates a reverse resistance change because of gas species. The n‐type and p‐type behavior determined through the altered direction of the charge carrier plays a dominant role in the conduction response.^39^, ^40^ Different conductivity types of graphene‐based sensors were reported in previous experimental studies, but no specific trend was observed.^38^ Based on our DFT calculations, we infer that H_2_S exhibits an n‐type behavior with electron depletion on itself. An increase in resistance was further observed in the sensing experiment, indicating the n‐type sensing nature of the Au‐modified graphene layer. In DFT calculations, SOF_2_ presents a p‐type behavior with electron withdrawing capability, in which electrons can be removed from the adsorbent surface. This phenomenon results in decreased resistance, thereby confirming that Au‐modified GrF displays a p‐type sensing behavior. The corresponding correlation between sensing performance and nature of carriers for Au‐modified GrF was obtained under our experiment conditions and illustrated in **Figure** **10**.

## 3 Conclusions

Detection of H_2_S and SOF_2_, which are two types of SF_6_ decomposed gases, has gained importance because they are significantly related to insulation faults in power equipment. As current detection methods suffer from online monitoring shortage, electrochemistry sensors could be a promising technology for online detection.

Pure graphene films and graphene films incorporated with AuNPs were fabricated as gas sensors through deposition–precipitation method. Sensing experiments conducted at room temperature demonstrated that Au‐modified GrF is a promising material for developing H_2_S and SOF_2_ selective sensors because it yields opposite responses for these gases. The interaction between the Au‐modified GrF surface and these gases was simulated through first‐principles calculations. The results reveal that the opposite responses observed in experiments could be due to the charge‐transfer differences related to the interfacial interaction within gases and Au‐modified GrF systems. Calculation and experimental findings consistently show n‐type H_2_S functions as an electron donor and p‐type SOF_2_ as an electron acceptor.

The results of sensing experiments further show the strong response of the Au‐modified GrF sensor for SOF_2_. This finding confirms the robustness and the ability to adsorb SOF_2_ of the sensor compared with the poor performance on pure graphene films. According to the results of DFT calculations, the typical chemisorption effect between decorated Au and SOF_2_ could be the main reason for this observation and the adsorption of the pristine graphene corresponds to physisorption dominated by van der Waals.

Our calculation results further show that the H_2_S chemisorption behavior endows Au‐modified GrF with a magnetic system, whereas SOF_2_ adsorption converts the system into a nonmagnetic one. This result may be utilized in designing novel magnetic sensing or switching devices, but requires further investigation through experimental methods.

## 4 Experimental Section

*Experimental Details*: Carboxylic functionalized graphene and rGO were purchased from XF NANO, Inc. (Nanjing, China). Dimethylformamide (DMF), acetone, and absolute ethyl alcohol were purchased from Huihuang Chemical Reagent Co., Ltd (Chongqing, China). NaBH_4_ and HAuCl_4_ were purchased from Aladdin Chemistry Co., Ltd. All chemicals in this work were of analytical grade and used without further purification, and double‐distilled water was used throughout the experiments.

Au‐modified GrF was synthesized through the following chemical reduction procedures.^29^ Carboxyl functionalized graphene (1 mg) was dispersed in HAuCl_4_ (5 mL, 1 × 10^−3^ m) solution as a precursor under constant sonication for 40 min to reach a stable colloidal state. Briefly, NaBH_4_ (5 mL, 40 × 10^−3^ m) solution was added dropwise to the colloid solution and the solution was vigorously stirred for 30 min. The solution was then subjected to centrifugal separation, and products were collected and washed with distilled water several times. The washed products were dried in a vacuum oven at 60 °C for 12 h to obtain Au‐modified GrF. Au‐modified GrF (5 mg) powder was ultrasonically dispersed in DMF solution (200 mL) for 30 min to achieve good dispersibility.

**Figure 11:**
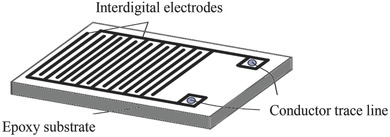
Schematic structural view of a planar sensor.

Sensors were fabricated using Au‐modified GrF through LBL deposition.^41^ Responses were measured by monitoring surface resistance changes in a pressure‐tight system. For the planar sensor depicted in **Figure** **11**, copper electrodes were inter‐digitally etched on epoxy resin with ≈30 μm thick foil and 0.2 mm electrode gap. The prepared Au‐modified GrF solution was continuously dispersed on the substrate and then dried until the desired initial surface resistance values, which translate to the formation of uniform, dense, and smooth deposited films, were achieved. The fabricated Au‐modified GrF sensor was used for detection. A sensing element was placed in an autonomous sealed chamber connected to an electrochemical analyzer. As the initial vacuum resistance stability is a prerequisite for gas detection experiment, responses were measured at room temperature and repeated several times to obtain reliable results.

X‐ray diffraction (XRD) patterns were recorded on a Rigaku D/Max‐1200X using Cu Kα radiation (*λ* = 0.15418 nm) at 40 kV and 30 mA. Wide‐angle XRD patterns were collected at a scanning speed of 10° per minute over the 2*θ* range of 5° to 100°. Raman spectra were determined using a Renishaw inVia Raman microscope configured with a 532 nm wavelength laser and full‐range grating. Scanning electron microscopy (SEM) images were recorded with a Zeiss Auriga Focus Ion Beam/Field‐Emission SEM dual‐cross system operated at 30 kV and 2 nA. The samples were directly exfoliated from the planar sensor. High‐resolution transmission electron microscopy (HRTEM) combined with energy dispersive spectroscopy (EDS) images was recorded with an FEI Tecnai G2 F20 S‐TWIN operated at 200 kV. X‐ray photoelectron spectroscopy (XPS) was performed on a Thermo ESCALAB 250Xi spectrometer with Al Kα (1486.6 eV) radiation. All measurements were conducted under ambient condition.

*Theoretical Methods*: To determine the role of van der Waals, we performed theoretical calculations by using dispersion‐corrected DFT (DFT‐D) provided by the DMol^3^ code. The exchange and correlation energies included were identified through a generalized gradient approximation in revised Perdew–Burke–Ernzerhof (PBE) format.^42^, ^43^, ^44^ Core treatment, in which core electrons are replaced by a single effective potential, was conducted with DFT semi‐core pseudopods to evaluate relativistic effects. To simulate a 2D graphene sheet, we modeled a supercell comprising 6 × 6 units (consisting of 72 atoms) in the XY plane. A vacuum region of 20 Å in the Z direction was also adopted to prevent the interaction between adjacent layers. The *k*‐point mesh was increased to 6 × 6 × 1 for the Brillouin‐zone integration to obtain accurate results. All calculations were performed in a spin‐unrestricted manner. The convergence tolerance of energy was set at 1.0 × 10^−6^ Ha in geometry optimization.

## Supporting information

As a service to our authors and readers, this journal provides supporting information supplied by the authors. Such materials are peer reviewed and may be re‐organized for online delivery, but are not copy‐edited or typeset. Technical support issues arising from supporting information (other than missing files) should be addressed to the authors.

Supplementary
