# Supplementary material for: Experimental Sensing and Density Functional Theory Study of H2S and SOF2 Adsorption on Au‐Modified Graphene
Source: Adv Sci (Weinh). 2015 Sep 10;2(11):1500101. doi: 10.1002/advs.201500101 (PMC5049619; doi:10.1002/advs.201500101)
Supplement: Supplementary file 1 — Supplementary [file ADVS-2-0e-s001.pdf]

Copyright WILEY-VCH Verlag GmbH & Co. KGaA, 69469 Weinheim, Germany, 2015.

ADVANCED SCIENCE
Open Access

Supporting Information

for *Adv. Sci.*, DOI: 10.1002/advs.201500101

Experimental Sensing and Density Functional Theory Study of H_2S and SOF_2 Adsorption on Au-Modified Graphene

Xiaoxing Zhang, Lei Yu, Xiaoqing Wu, and Weihua Hu*

SUPPORTING INFORMATION

In order to find out the most stable and possible position that Au atom forms on the graphene surface, the formation energy, which is short for $E_{form}(C\rightarrow Au)$, of every modification model was calculated in DFT study. The $E_{form}(C\rightarrow Au)$ was calculated through the principle formula:

$$E_{form}(C \rightarrow Au) = E_{tot}(Au - functionalized) - E_{tot}(pristine) + \mu_C - \mu_{Au} \qquad \text{(S.1)}$$

where $E_{tot}(Au\text{-}functionalized)$ represents for the total energy of Au modified graphene; $E_{tot}(pristine)$ represents for the total energy of pure graphene; μ_C and μ_{Au} are the energy of single atom in individual crystal.

Table S1 gives out the formation energies of H, B and T site models. The T site model is proved to be with the lowest energy, indicating that the T site model is the most possible position for Au.

Table S1 Formation energy of Au-modified graphene

Position	H site	B site	T site
$E_{form}(C\rightarrow Au)$ (eV)	1.71	1.42	0.89
